# Acute Pancreatitis After Percutaneous Metallic Stent Insertion for Malignant Biliary Obstruction: A Retrospective 2-Center Study

**DOI:** 10.5152/tjg.2023.22442

**Published:** 2023-09-01

**Authors:** Chen Xu, Guo-xiong Xu, Sheng Liu, Hai-bin Shi, Wei-zhong Zhou

**Affiliations:** 1 Department of Intervention Radiology, Suzhou Municipal Hospital Affiliated to Nanjing Medical University, Suzhou, China; 2 Department of Interventional Radiology, The First Affiliated Hospital of Nanjing Medical University, Nanjing, China

**Keywords:** Malignant biliary obstruction, percutaneous transhepatic biliary stenting, acute pancreatitis

## Abstract

**Background/Aims:**

The current study investigated the incidence, risk factors, and outcomes of acute pancreatitis after percutaneous transhepatic biliary stenting for malignant biliary obstruction.

**Materials and Methods:**

From March 2016 to May 2020, a total of 425 patients who underwent percutaneous transhepatic biliary stenting for malignant biliary obstruction were included in this 2-center study. After the procedure, we analyzed the incidence, risk factors, and outcomes of acute pancreatitis.

**Results:**

On follow-up, 79 (18.6%) patients showed increased serum amylase levels, of whom 41 (9.6%) developed pancreatitis. On binary logistic regression analysis, stent across the duodenal papilla (odds ratio = 8.54; 95% CI = 3.54-20.62;* P* < .001) and visualization of the pancreatic duct (odds ratio = 9.87; 95% CI = 4.67-20.86;* P* < .001) were significant risk factors of pancreatitis after the procedure. Using conservative therapy, all patients were successfully managed at a mean of 3.5 days (range 1-6 days), and no severe pancreatitis happened.

**Conclusion:**

Acute pancreatitis is a relatively common complication after percutaneous transhepatic biliary stenting. Stent across the duodenal papilla and visualization of the pancreatic duct are independent risk factors.

**Main Points**

- Acute pancreatitis is a relatively common complication after percutaneous transhepatic biliary stenting.
- The current study is a retrospective two- center study conducted on a cohort of 425 patients.
- Stent across the duodenal papilla and visualization of the pancreatic duct are independent risk factors.

## Introduction

Insertion of metallic stent has been recognized as an effective therapy for patients with malignant biliary obstruction (MBO).^1-4^ Generally, a stent can be placed endoscopically or percutaneously, and each approach possesses its own pros and cons.^5-9^

Acute pancreatitis is a life-threatening complication following the metallic biliary stent insertion.^10-12^ Compared with an endoscopic approach, pancreatitis after percutaneous transhepatic biliary stenting (PTBS) is scarcely investigated.^11,13^ Although several studies reported the risk factors of post-procedural pancreatitis, their results were inconsistent and limited by small sample sizes.^9,13,14^ Currently, the risk factors of pancreatitis after PTBS remain elusive and require further clarification.

The purpose of this study was to explore the incidence, risk factors, and outcomes of pancreatitis after PTBS for MBO.

## Materials and Methods

### Patients

**Figure 1.**
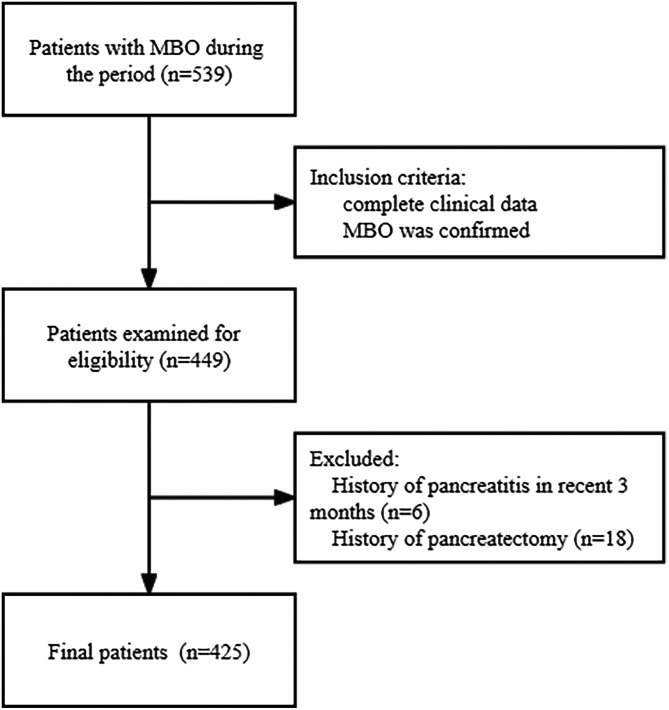
Study design. MBO, malignant biliary obstruction.

This retrospective, 2-center study was approved by the institutional review board of Nanjing Medical University (Approval No: 2020-SR- 200, Date: 2020.05.13), and verbal consent was obtained from the patients. Data of 539 MBO patients who underwent PTBS between March 2016 and May 2020 were reviewed. Inclusion criteria of this study included: (1) complete clinical data including laboratory indexes and imaging information and (2) MBO confirmed based on radiological and/or pathological findings. Exclusion criteria included: (1) patients with a history of pancreatitis in the recent 3 months at admission or (2) a history of pancreatectomy. A total of 425 consecutive patients were enrolled (Figure 1). Among them, 202 were men, while 223 were women. The mean age of the patients was 66.8 years (range 38-92 years). The diagnosis of the primary tumor was established following the radiological findings of 194 patients and pathology results of 231 patients. Notably, the primary cholangiocarcinoma accounted for 31.1%, hepatocellular carcinoma for 25.2%, gallbladder cancer for 19.5%, pancreatic cancer for 13.6, and the other metastases for 9.9%. The characteristics of patients are summarized in Table 1.

### Metallic Stent Insertion

**Figure 2.**
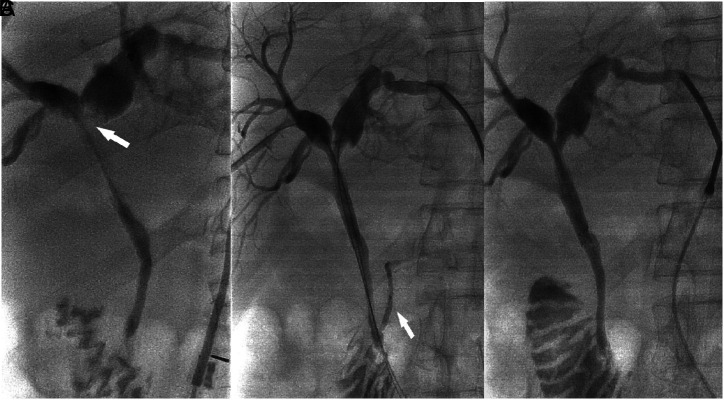
Male, 55 years old, cholangiocarcinoma. (A) Cholangiography before stent insertion, which demonstrated the Bismuth–Corlette type II malignant biliary obstruction (arrow). (B) Incidental visualization of the pancreatic duct (arrow). (C) Cholangiography after stent insertion. Two metallic uncovered stents (8 mm in diameter and 60 mm in length) were placed to cover the obstruction completely.

Before the procedure, all patients were required to fast for at least 12 h. Procedures were performed by 3 interventional radiologists from 2 centers with over 10 years of experience under fluoroscopic guidance. Biliary stent insertion was performed under local anesthesia with intramuscular lidocaine. The intrahepatic bile duct was punctured with a 21-gauge Chiba needle (Cook, Bloomington, Ind, USA). Upon successful puncture, a 0.018-inch guidewire was inserted, and thereafter a 4F introducer sheath (Neff Percutaneous Access Set, Cook) was introduced. Cholangiography was then executed to evaluate the obstruction site. A 0.035-inch guidewire was then advanced to the duodenum across the obstruction site with a 4F catheter. After measurement of the length of stricture, the stent was introduced over the guidewire and then deployed across the stricture to cover the bile duct approximately 1.5-2 cm distal and proximal to the obstruction to prevent tumor infiltration. If lesions were located within 3 cm of the duodenal papilla, the distal portion of the stent was placed across the papilla to bridge the duodenum. Stent patency was confirmed with repeat cholangiography. During the procedure, the external drainage tube (8F, Cook) was inserted in patients with infection, and the iodine-125 seeds (0.8 mci, Xinke, Shanghai, China) strand was inserted for intraluminal radiotherapy in 61 patients with their permission. The puncture approach was occluded with gel foam pledgets through a sheath (Figure 2). Three types of uncovered self-expanding metallic stent with a diameter of 8 mm and lengths from 60 to 100 mm were used in this study [E-Luminexx (Bard Peripheral Vascular, Tempe, Ariz, USA), S.M.A.R.T. (Cordis, Milpitas, Calif, USA), and Zilver (Cook, Bloomington, Ind, USA)]. Characteristics of the procedure are depicted in Table 2.

### Definition

Acute pancreatitis was diagnosed according to the Atlanta classification,^15^ which requires the presence of 2 or more of the following criteria: persistent abdominal pain along with vomiting and nausea; serum amylase levels of at least 3 times the upper limit of normal; and imaging findings including computed tomography (CT) or ultrasonography. The severity of acute pancreatitis was also evaluated following the Atlanta classification guideline.^15^ Mild pancreatitis was defined by the absence of organ failure and no local or systemic complications; moderate pancreatitis was defined as transient organ failure that resolved within 48 hours and/or local or systemic complications without persistent organ failure; and eventually, severe pancreatitis was delineated as persistent organ failure. Especially, hyperamylasemia was defined as a normal clinical condition at 24 hours after PTBS but with serum amylase levels elevated above the upper limit of normal.

### Follow-up

After the procedure, the serum amylase levels and clinical condition of all patients were monitored daily. Imaging examinations were used to evaluate acute pancreatitis, while the serum amylase levels were over 3 times upper the limit of normal without clinical symptoms. The first postoperative serum amylase levels were determined for 3 hours after the procedure. Patients with pancreatitis were treated with somatostatin (6 mg solved in 500 mL 0.9% sodium chloride solution) and fasting therapy, and no non-steroidal antiinflammatory drugs and fluid infusions as prophylaxis were performed.

### Risk Factors

The risk factors for the development of pancreatitis after PTBS were evaluated by analyzing the characteristics of patients and procedures. We collected the following variables: age and gender, primary tumor, underlying disease, previous biliary drainage, preoperative infection, level of stricture, hypersensitive-c-reactive-protein (hs-CRP), pancreas atrophy, time of the procedure, external drainage tube, iodine-125 seed strand, visualization of the pancreatic duct, stent length, stent type, stent number, and site of stents. Among the above variables, pancreas atrophy was defined as the thickness of the pancreatic parenchyma in the pancreatic body <10 mm and dilatation of the main pancreatic duct.^9,14^ Time of the procedure was defined as the time from a successful puncture to repeat cholangiography confirmed with stent patency. The level of the common bile duct (CBD) stricture was determined by preoperative CT images and cholangiography images. The level of upper CBD was defined as above the confluence of the cystic duct, below which represented the lower CBD and both involved represented the whole CBD.

### Statistical Analysis

Normally distributed data are expressed as means ± SD, while non-normally distributed data are presented as the interquartile range. Differences in measurement data were evaluated using the *t*-test or Mann–Whitney *U*-test when appropriate. Categorical data were compared using the chi-square or Fisher’s exact tests as appropriate and presented as frequencies. The significant variables in univariate analysis were subsequently entered into the binary logistic regression analysis method to seek the independent predictor of acute pancreatitis. Statistical analyses were conducted with the use of Statistical Package for Social Sciences software, version 19.0 (SPSS Inc., Chicago, IL, USA). We calculated odds ratio (OR) with 95% CIs, while values of *P* < .05 were considered statistically significant.

## Results

### Frequency and Clinical Prognosis

**Figure 3.**
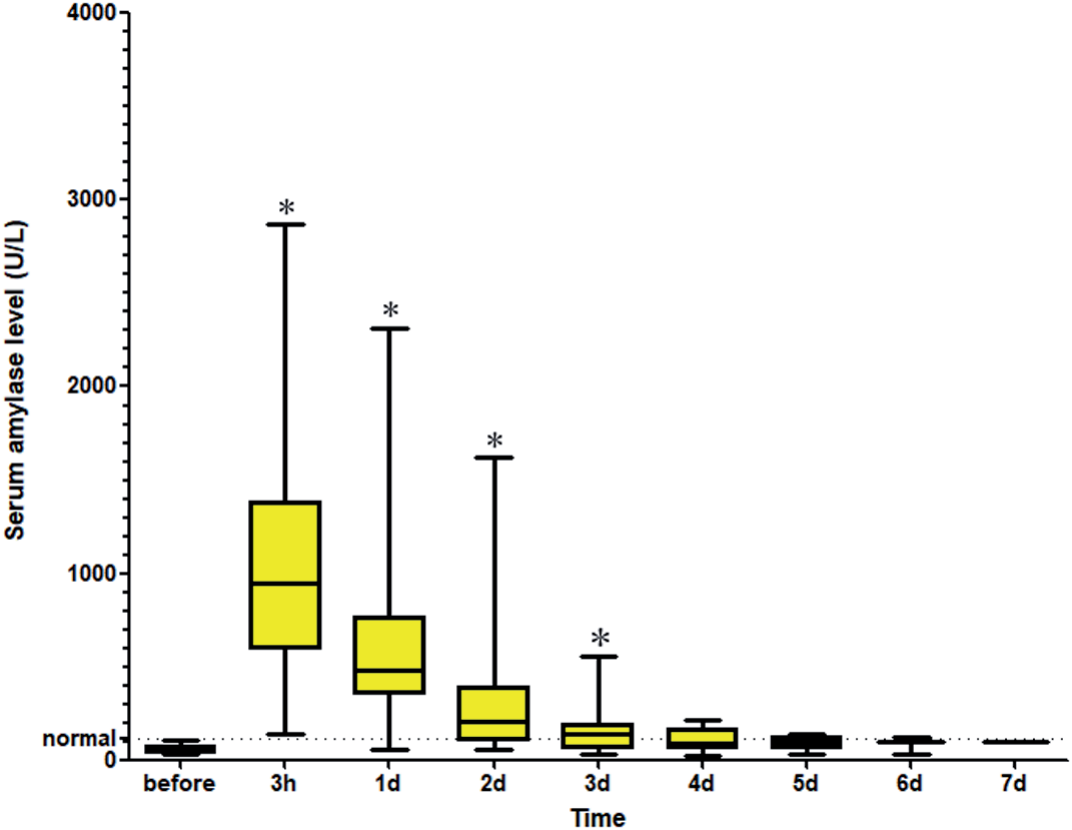
The serum amylase level of patients with acute pancreatitis. *P < .05.

The technical success of PTBS was 100%. After the procedure, 79 (18.6%) patients presented increased levels of serum amylase, while 41 (9.6%) patients were diagnosed with pancreatitis. Among these patients, the median serum amylase level increased significantly from 52.4 (30.2) U/L to 943.7 (776.6) U/L (*z* = –7.60, *P* < .001) after the procedure and rapidly decreased within the first 3 days for most patients (Figure 3).

All patients with pancreatitis were successfully managed at a mean of 3.5 days (range 1-6 days) using conservative therapy. Of the 41 patients, only 1 patient presented moderate pancreatitis and recovered within 48 hours, and no severe pancreatitis occurred. Notably, while the 3-hour serum amylase level was ≥1000 IU/L, time for patients to recover was considerably longer compared with others (3.9 ± 1.1 vs. 3.1 ± 1.3 days, *t* = 2.17,* P* = .036).

### Risk Factors

On univariate analysis of risk factors, stent across the duodenal papilla and visualization of the pancreatic duct might be associated with pancreatitis after PTBS (Table 3) and were subsequently confirmed by the binary logistic regression analysis (Table 4). This implies that the odds of developing pancreatitis were more than 8 times higher for stent insertion across the duodenal papilla than above the papilla (OR = 8.54; 95% CI = 3.54-20.62;* P* < .001). In addition, the odds of developing pancreatitis were nearly 10 times higher in patients with visualization of the pancreatic duct (OR = 9.87; 95% CI = 4.67-20.86; *P* < .001).

## Discussion

The results of this study indicate that 79 (18.6%) patients showed increased levels of serum amylase after PTBS, while pancreatitis was observed in 41 (9.6%) patients, and the incidence of pancreatitis after PTBS in this study agrees with previous reports of 3.5%-25.0%.^7-10,13^ We further discovered that a high 3-hour serum amylase level (≥1000 IU/L) might reflect a longer hospital stay and poor prognosis (*P* = .036).

The risk factors of pancreatitis after PTBS remain largely unknown. Takeda et al^16^ reported that high pancreatic volume is a risk factor for pancreatitis after endoscopic metal stent placement. Besides, Kawakubo et al^6^ suggested that non-pancreatic cancer is one of the risk factors for pancreatitis due to normal pancreatic function. However, in this research, it was not statistically associated with pancreatitis following PTBS. This may be explained by the different approaches and the fact that the proportion of pancreatic cancer was 13.6% in this study, which was far lower than the 52%-84% found in previous reports.^6,17-22^ Therefore, future studies are needed to identify whether pancreatic cancer reduces the incidence of post-PTBS pancreatitis.

In the present investigation, the incidence of pancreatitis following the procedure was markedly higher for the stent insertion across the papilla compared with that of above the papilla. Jo and Park^7^ reported that the incidence of pancreatitis was higher in patients with stent placement across the papilla (25.0%) than above the papilla (4.1%). Moreover, Tarnasky et al^23^ revealed that compression of the pancreatic duct orifice due to the medial defection of biliary stents leads to pancreatitis. Considering previous studies showed that stent placement above the duodenal papilla does not increase the development of stent occlusion or cholangitis,^24,25^ it could be more reasonable for biliary stent insertion above the papilla if possible.

Furthermore, during the procedure, visualization of the pancreatic duct was shown to be largely associated with pancreatitis after PTBS. This may arise from the high pressure of the pancreatic duct and chemical stimulation.^26,27^ In this regard, the contrast medium should thus be injected carefully with the appropriate pressure.

Despite these intriguing results, this study has some inherent limitations. First, data collection was performed retrospectively, which may affect the reliability of the evaluated data. Second, sometimes it is difficult to distinguish atypical pancreatitis pain from post-procedure pain; therefore, it can be possible that cases of undiagnosed pancreatitis may have been presented in the cohort. Finally, all stents in this study were uncovered with the same diameter of 8 mm; thus, the risk factors of stent coverings and diameters require further exploration.

## Conclusion

In summary, our findings revealed that pancreatitis is a relatively common complication after PTBS for MBO with an incidence of 9.6%. Notably, stents across the papilla and visualization of the pancreatic duct are independent risk factors.

## Figures and Tables

**Table 1.** Patient Characteristics

Classification	n (%)
Gender	
Male	202 (47.5)
Female	223 (52.5)
Age, years, mean (range)	66.8 (38-92)
Primary tumor	
Cholangiocarcinoma	132 (31.1)
Hepatocellular carcinoma	107 (25.2)
Gallbladder cancer	83 (19.5)
Pancreatic cancer	58 (13.6)
Others	45 (9.9)
Underlying disease	
Hypertension	165 (38.8)
Diabetes mellitus	83 (19.5)
Cardiovascular disease	42 (10.1)
Previous biliary drainage	177 (41.6)
Preoperative infection	127 (29.9)
Level of stricture	
Upper CBD	196 (46.1)
Lower CBD	157 (36.9)
Whole CBD	72 (17.0)
hs-CRP, mg/L, median (IQR)	7.9 (3.4-17.2)
Pancreas atrophy	23 (5.4)

*CBD, common bile duct; hs-CRP, hypersensitive C-reactive protein; IQR, inter-quartile range.

**Table 2.** Procedure Characteristics

Classification	n (%)
Procedure time, min	
≥60	87 (20.5)
<60	338 (79.5)
External drainage tube	234 (55.1)
Iodine-125 seed strand	61 (14.4)
Visualization of the pancreatic duct	66 (15.5)
Stent length, cm	
6	154 (36.3)
8	202 (47.5)
10	69 (16.2)
Stent stype	
Bard	177 (41.6)
Cordis	152 (35.8)
Cook	96 (22.6)
Number of stents	
One	282 (66.4)
Multiple	143 (33.6)
Distal portion of the stent	
Across the papilla	171 (40.2)
Above the papilla	254 (59.8)

*Stent length was evaluated as the maximum length while multiple stents insertion.

**Table 3.** Univariate Analysis of Risk Factors for Acute Pancreatitis After PTBS

Variable	Pancreatitis (-) (n = 384)	Pancreatitis (+) (n = 41)	*P*
Gender			.413
Male	185 (48.2)	17 (41.5)	
Female	199 (51.8)	24 (58.5)	
Age, years			.798
≥60	288 (75.0)	30 (73.2)	
<60	96 (25.0)	11 (26.8)	
Hypertension			.757
Yes	150 (39.1)	15 (36.6)	
No	234 (60.9)	26 (63.4)	
Diabetes mellitus			.406
Yes	77 (20.1)	6 (14.6)	
No	307 (79.9)	35 (85.4)	
Cardiovascular disease			.602
Yes	37 (9.6)	5 (12.2)	
No	347 (90.4)	36 (87.8)	
Diagnosis			.846
Pancreatic cancer	52 (13.5)	6 (14.6)	
Non-pancreatic cancer	332 (86.5)	35 (85.4)	
Previous biliary drainage			.521
Yes	158 (41.1)	19 (46.3)	
No	226 (58.9)	22 (53.7)	
Preoperative infection			.530
Yes	113 (29.4)	14 (34.1)	
No	271 (70.6)	27 (65.9)	
Level of stricture			.435
Upper CBD	139 (36.2)	18 (43.9)	
Lower CBD	181 (47.1)	15 (36.6)	
Whole CBD	64 (16.7)	8 (19.5)	
hs-CRP, mg/L			.935
≥8	138 (35.9)	15 (36.6)	
<8	246 (64.1)	26 (63.4)	
Pancreas atrophy			.602
Yes	22 (5.7)	1 (2.4)	
No	362 (94.3)	40 (97.6)	
Operation time, min			.288
≥60	76 (19.8)	11 (26.8)	
<60	308 (80.2)	30 (73.2)	
External drainage tube			.395
Yes	170 (44.3)	21 (51.2)	
No	214 (55.7)	20 (48.8)	
Iodine-125 seed strand			.572
Yes	53 (13.8)	7 (17.1)	
No	330 (86.2)	34 (82.9)	
Visualization of the pancreatic duct			<.001
Yes	43 (11.2)	23 (56.1)	
No	341 (88.8)	18 (43.9)	
Stent length, cm			.274
6	141 (36.7)	13 (31.7)	
8	178 (46.4)	24 (58.5)	
10	65 (16.9)	4 (9.8)	
Stent stype			.419
Bard	159 (41.4)	18 (43.9)	
Cordis	135 (35.2)	17 (41.5)	
Cook	90 (23.4)	6 (14.6)	
Number of stents			.443
One	257 (66.9)	25 (61.0)	
Multiple	127 (33.1)	16 (39.0)	
Distal portion of the stent			<.001
Across the papilla	137 (35.7)	34 (82.9)	
Above the papilla	247 (64.3)	7 (17.1)	

**Table 4.** Logistic Regression Analysis of the Risk Factors for Pancreatitis After PTBS

	*B*	*P*	*OR*	*95% CI*
Visualization of the pancreatic duct	2.29	<.001	9.87	4.67-20.86
Stent across the papilla	2.15	<.001	8.54	3.54-20.62
